# A Mapping of Drug Space from the Viewpoint of Small Molecule Metabolism

**DOI:** 10.1371/journal.pcbi.1000474

**Published:** 2009-08-21

**Authors:** James Corey Adams, Michael J. Keiser, Li Basuino, Henry F. Chambers, Deok-Sun Lee, Olaf G. Wiest, Patricia C. Babbitt

**Affiliations:** 1 Graduate Program in Pharmaceutical Sciences and Pharmacogenomics, University of California, San Francisco, California, United States of America; 2 Graduate Program in Bioinformatics, University of California, San Francisco, California, United States of America; 3 San Francisco General Hospital, University of California San Francisco, San Francisco, California, United States of America; 4 Center for Complex Network Research and Departments of Physics, Biology, and Computer Science, Northeastern University, Boston, Massachusetts, United States of America; 5 Center for Cancer Systems Biology, Dana-Farber Cancer Institute, Boston, Massachusetts, United States of America; 6 Department of Natural Medical Sciences, Inha University, Incheon, Korea; 7 Department of Chemistry and Biochemistry, University of Notre Dame, Notre Dame, Indiana, United States of America; 8 Department of Bioengineering and Therapeutic Sciences, University of California, San Francisco, California, United States of America; 9 Department of Pharmaceutical Chemistry, University of California, San Francisco, California, United States of America; 10 California Institute for Quantitative Biosciences, University of California, San Francisco, California, United States of America; University of California San Diego, United States of America

## Abstract

Small molecule drugs target many core metabolic enzymes in humans and pathogens, often mimicking endogenous ligands. The effects may be therapeutic or toxic, but are frequently unexpected. A large-scale mapping of the intersection between drugs and metabolism is needed to better guide drug discovery. To map the intersection between drugs and metabolism, we have grouped drugs and metabolites by their associated targets and enzymes using ligand-based set signatures created to quantify their degree of similarity in chemical space. The results reveal the chemical space that has been explored for metabolic targets, where successful drugs have been found, and what novel territory remains. To aid other researchers in their drug discovery efforts, we have created an online resource of interactive maps linking drugs to metabolism. These maps predict the “effect space” comprising likely target enzymes for each of the 246 MDDR drug classes in humans. The online resource also provides species-specific interactive drug-metabolism maps for each of the 385 model organisms and pathogens in the BioCyc database collection. Chemical similarity links between drugs and metabolites predict potential toxicity, suggest routes of metabolism, and reveal drug polypharmacology. The metabolic maps enable interactive navigation of the vast biological data on potential metabolic drug targets and the drug chemistry currently available to prosecute those targets. Thus, this work provides a large-scale approach to ligand-based prediction of drug action in small molecule metabolism.

## Author Summary

All humans, plants, and animals use enzymes to metabolize food for energy, build and maintain the body, and get rid of toxins. Drugs used to clear infections or cure cancer often target enzymes in bacteria or cancer cells, but the drugs can interfere with the proper function of human enzymes as well. Recent studies have mapped drugs to enzymes and many other targets in humans and other organisms, but have not focused on metabolism. In this study, we present a new method to predict what enzymes drugs might affect based on the chemical similarity between classes of drugs and the natural chemicals used by enzymes. We have applied the method to 246 known drug classes and a collection of 385 organisms (including 65 National Institutes of Health Priority Pathogens) to create maps of potential drug action in metabolism. We also show how the predicted connections can be used to find new ways to kill pathogens and to avoid unintentionally interfering with human enzymes.

## Introduction

Drug developers have long mined small molecule metabolism for new drug targets and chemical strategies for inhibition. The approach leverages the “chemical similarity principle” [1] which states that similar molecules likely have similar properties. Applied to small molecule metabolism, this principle has motivated the search for enzyme inhibitors chemically similar to their endogenous substrates. The approach has yielded many successes, including antimetabolites such as the folate derivatives used in cancer therapy and the nucleoside analog pro-drugs used for antiviral therapy. However, drug discovery efforts also frequently falter due to unacceptable metabolic side-effect profiles or incomplete genomic information for poorly characterized pathogens [2]–[4].

With the recent availability of large datasets of drugs and drug-like molecules, computational profiling of small molecules has been performed to create global maps of pharmacological activity. This in turn provides a larger context for evaluation of metabolic targets. For example, Paolini et al. [5] identified 727 human drug targets associated with ligands exhibiting potency at concentrations below 10 µM, thereby creating a polypharmacology interaction network organized by the similarity between ligand binding profiles. Keiser et al. [6] organized known drug targets into biologically sensible clusters based solely upon the bond topology of 65,000 biologically active ligands. The results revealed new and unexpected pharmacological relationships, three of which involved GPCRs and their predicted ligands that were subsequently confirmed *in vitro*. Cleves et al. [7] also rationalized several known drug side effects and drug-drug interactions based upon three-dimensional modeling of 979 approved drugs. However, despite the clear rationale and past successes in applying ligand-based approaches to drug discovery, global mapping between drugs and small molecule metabolism, the goal of this study, has been hindered by both methodological challenges and incomplete genomic information. The relatively recent availability of metabolomes for numerous organisms allows a fresh look on a large scale [8]–[13].

In this work, we link the chemistry of drugs to the chemistry of small molecule metabolites to investigate the intersection between small molecule metabolism and drugs. The Similarity Ensemble Approach (SEA) [6] was used to link metabolic reactions and drug classes by their chemical similarity, measured by comparing bond topology patterns between sets of molecules. Two types of molecule sets are used in this work. The first comprises drug-like molecules known to act at a specific protein target, and the second comprises the known substrates and products of an enzymatic reaction. While this approach is complementary to target and disease focused methods [5], [14]–[23], neither protein structure nor sequence information is used in the comparisons. Thus, these links provide an orthogonal view of metabolism based only upon the chemical similarity between existing drug classes and endogenous metabolites.

To provide the results in the context of metabolism, drug “effect-space” maps were also created. For each of the 246 drug classes investigated in this work, effect-space maps enable visualization of the chemical similarities between drugs and metabolites painted onto human metabolic pathways, allowing a unique assessment of potential drug action in humans. In addition, to aid target discovery in pathogens, 385 species-specific effect-space maps were created to show the predicted effect-space of currently marketed drugs, painted onto metabolic pathways representing target reactions in model organisms and pathogens. Examples of these maps are provided below and their applications in predicting drug action, toxicity, and routes of metabolism are discussed. To enable facile exploration of the drug-metabolite links established by this analysis, interactive versions of both sets of maps are available at http://sea.docking.org/metabolism.

Finally, using methicillin-resistant *Staphylococcus aureus* (MRSA), a major pathogen causing both hospital- and community-acquired infections that is resistant to at least one of the antibiotics most commonly used for treatment [24]–[28] as an example, we show by retrospective analysis the use of species-specific maps for discovery and evaluation of drug targets. This also illustrates how additional types of biological information can be incorporated to enhance the value of these analyses.

## Results

### Drug-metabolite links reproduce known drug-target interactions

**Figure 1:**
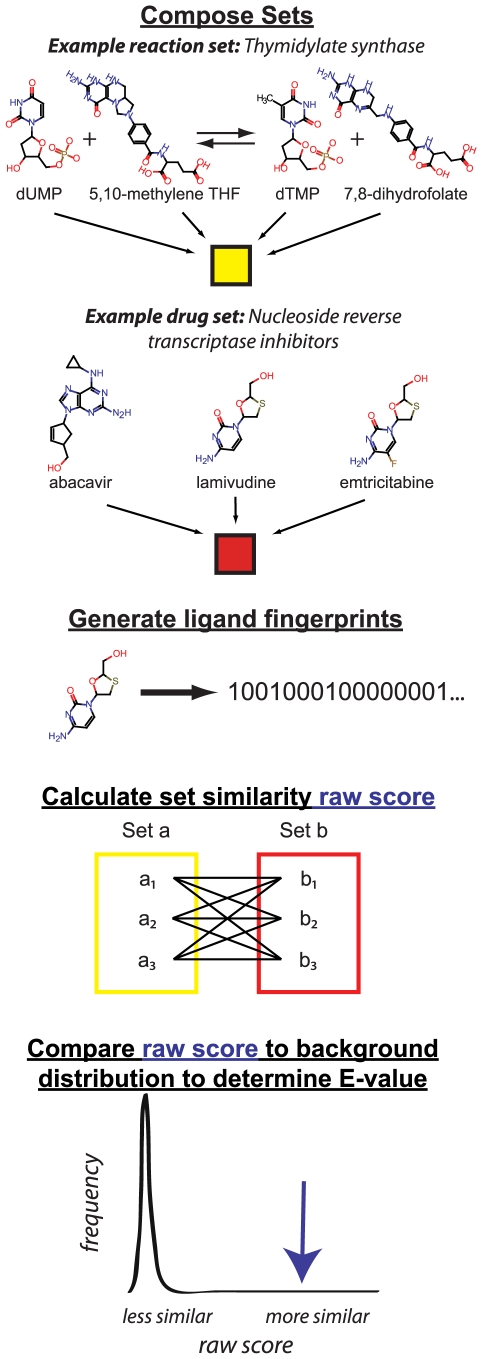
Similarity Ensemble Approach (SEA). SEA compares groups of ligands based upon bond topology. Example ligand sets include the thymidylate synthase reaction set, composed of the reaction substrates and products, and the nucleotide reverse transcriptase inhibitor (NRTI) drug set, which includes known inhibitors of the nucleoside reverse transcriptase enzyme. Fingerprints representing the bond topology of each molecule are generated. Raw scores between sets are calculated based upon Tanimoto coefficients between fingerprints for all molecule pairs. Finally, the raw scores are compared to a background distribution to determine the expectation value (E) representing the chemical similarity between sets. See Methods for further details.

To evaluate the chemical similarity between drug classes and metabolic reactions, links between sets of metabolic ligands and sets of drugs were generated according to SEA (**Figure 1**) [6]. The similarity metric consists of a descriptor, represented by standard two-dimensional topological fingerprints, and a similarity criterion, the Tanimoto coefficient (Tc). Expectation (E) values were calculated for each set pair by comparing the raw scores to a background distribution generated using sets of randomly selected molecules (see **Methods** for further details). To represent metabolic ligand sets, the MetaCyc database, which includes enzymes from more than 900 different organisms catalyzing over 6,000 reactions, was used [12]. The substrates and products of each enzymatic reaction were combined to form a reaction set, each of which was required to contain at least two unique compounds (**Datasets S1** and **S2**). Ubiquitous molecules called common carriers, which frequently play critical roles in reaction chemistry but do not distinguish the function of a specific enzyme, were removed, leaving a total of 5,056 reactions involving 4,998 unique compounds. To represent drugs, a subset of 246 targets of the MDL Drug Data Report (MDDR) collection, which annotates ligands according to the targets they modulate, was used (**Datasets S3** and **S4**) [30]. These sets contain 65,241 unique ligands with a median and mean of 124 and 289 ligands per target, respectively. Overall, 246 drug versus 5,056 reaction set comparisons involving 1.39×10^9^ pairwise comparisons were made.

Although drugs and metabolites typically differ in their physiochemical properties, significant and specific similarity links nonetheless emerged. Using SEA at an expectation value cutoff of E = 1.0×10^−10^, a previously reported cutoff for significance [6], 54% (132 of 246) of drug sets link to an average of 43.7 (median = 10) or 0.9% of metabolic reactions. None of the remaining 46% (114 of 246) of drug sets link to any metabolic reaction sets. For instance, while the α-glucosidase drug set links to the α-glucosidase reaction (E = 1.00×10^−51^), the thrombin inhibitor drug set does not link significantly with any metabolic reaction. The thrombin inhibitor drug set targets the serine protease thrombin, which does not participate in small molecule metabolism, but rather plays a role in the coagulation signaling cascade. Likewise, 40% (2,044 of 5,056) of metabolic reactions hit an average of 2.8 (median = 2) or 1.1% of drug sets at expectation value E = 1.0×10^−10^ or better. For instance, the metabolite set for retinal dehydrogenase reaction set links, as expected, to the retinoid drugs at E = 3.05×E^−98^, but the valine decarboxylase reaction, which is not an MDDR drug target, does not link significantly to any drug sets. These strikingly similar results suggest both broad coverage (54% of drug sets and 40% of metabolite sets) and specificity (single sets link to just 0.9% of metabolite sets and 1.1% of drug sets, respectively). For full results, see **Dataset S5**.

**Figure 2:**
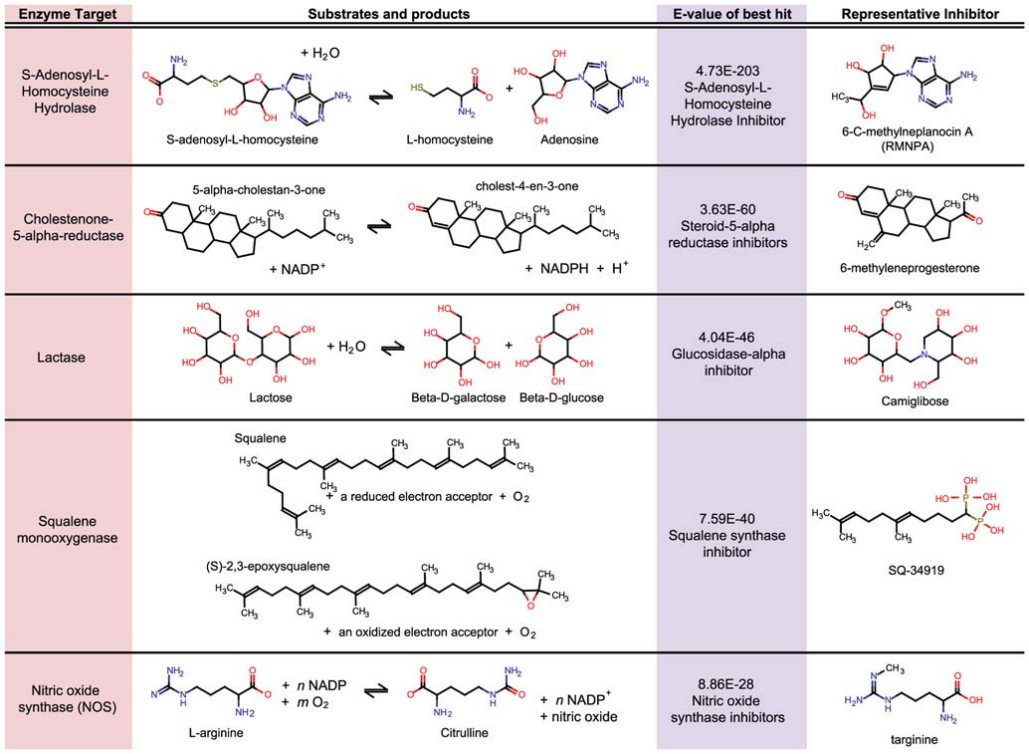
Selected best hits between MetaCyc reaction sets and MDDR drug sets.

To determine the utility of the method for recovery of known drug-target interactions, it was hypothesized that chemical similarity between MetaCyc reaction sets and corresponding MDDR drug sets could specifically recover the known drug-target interactions. The 246 MDDR drug set targets include 62 enzymes that could be mapped to MetaCyc via the Enzyme Commission (EC) number [31] describing the overall reaction catalyzed [32]. The results show that all 62 reaction sets for these targets link to at least one MDDR drug set. The majority of best hits (42 out of 62) were found at expectation values of E = 1.0×10^−10^ or better (**Table 1**). At expectation values better than E = 1.0×10^−25^, 61% (19 of 31) of best hits recover either the specific known target or another enzyme in the same pathway. Examples of specific compounds linked by this analysis are given in **Figure 2** for a selected group of these best-scoring hits.

**Table 1:** Metabolic enzyme targets and their best links to MDDR.

Enzyme Target^a^	EC#	Best Hit MDDR Drug Set	Best Hit E-value
*Adenosine kinase*	*2.7.1.20*	*S-Adenosyl-L-Homocysteine Hydrolase Inhibitor*	*4.38E-288*
Adenosylmethionine decarboxylase	4.1.1.50	S-Adenosyl-L-Homocysteine Hydrolase Inhibitor	2.71E-216
*Thromboxane-A synthase*	*5.3.99.5*	*Prostaglandin*	*1.66E-204*
**Adenosylhomocysteinase**	**3.3.1.1**	**S-Adenosyl-L-Homocysteine Hydrolase Inhibitor**	**4.73E-203**
Adenosine deaminase	3.5.4.4	Adenosine (A1) Agonist	7.69E-159
**Thymidine kinase**	**2.7.1.21**	**Thymidine Kinase Inhibitor**	**3.19E-151**
Dihydrofolate reductase	1.5.1.3	Glycinamide Ribonucleotide Formyltransferase Inhibitor	1.02E-134
Catechol O-methyltransferase	2.1.1.6	S-Adenosyl-L-Homocysteine Hydrolase Inhibitor	4.67E-127
**Prostaglandin-endoperoxide synthase**	**1.14.99.1**	**Prostaglandin**	**8.57E-110**
Purine-nucleoside phosphorylase	2.4.2.1	Adenosine (A1) Agonist	8.35E-105
Ribose-phosphate pyrophosphokinase	2.7.6.1	S-Adenosyl-L-Homocysteine Hydrolase Inhibitor	4.33E-91
**Phosphoribosylglycinamide formyltransferase**	**2.1.2.2**	**Glycinamide Ribonucleotide Formyltransferase Inhibitor**	**1.55E-82**
*Phosphoribosylaminoimidazolecarboxamide formyltransferase*	*2.1.2.3*	*Glycinamide Ribonucleotide Formyltransferase Inhibitor*	*9.12E-80*
3′,5′-cyclic-nucleotide phosphodiesterase	3.1.4.17	S-Adenosyl-L-Homocysteine Hydrolase Inhibitor	1.23E-77
**Thymidylate synthase**	**2.1.1.45**	**Thymidylate Synthetase Inhibitor**	**2.54E-75**
*Steryl-sulfatase*	*3.1.6.2*	*Aromatase Inhibitor*	*4.90E-62*
Guanylate cyclase	4.6.1.2	Purine Nucleoside Phosphorylase Inhibitor	2.68E-60
**Cholestenone 5-alpha-reductase**	**1.3.1.22**	**Steroid (5alpha) Reductase Inhibitor**	**3.63E-60**
*Steroid 17-alpha-monooxygenase*	*1.14.99.9*	*Steroid (5alpha) Reductase Inhibitor*	*1.37E-58*
RNA-directed DNA polymerase	2.7.7.49	S-Adenosyl-L-Homocysteine Hydrolase Inhibitor	1.06E-52
**Alpha-glucosidase**	**3.2.1.20**	**Glucosidase (alpha) Inhibitor**	**1.00E-51**
**Farnesyl-diphosphate farnesyltransferase**	**2.5.1.21**	**Squalene Synthase Inhibitor**	**2.12E-46**
*Beta-galactosidase*	*3.2.1.23*	*Glucosidase (alpha) Inhibitor*	*4.04E-46*
Sterol esterase	3.1.1.13	Phospholipase A2 Inhibitor	3.18E-44
*Leukotriene-A4 hydrolase*	*3.3.2.6*	*Prostaglandin*	*5.16E-40*
*Squalene monooxygenase*	*1.14.99.7*	*Squalene Synthase Inhibitor*	*7.59E-40*
Ribonucleoside-diphosphate reductase	1.17.4.1	S-Adenosyl-L-Homocysteine Hydrolase Inhibitor	2.47E-38
**3-hydroxyanthranilate 3,4-dioxygenase**	**1.13.11.6**	**3-Hydroxyanthranilate Oxygenase Inhibitor**	**1.14E-33**
**Dihydroorotase**	**3.5.2.3**	**Dihydroorotase Inhibitor**	**2.25E-32**
**Nitric-oxide synthase**	**1.14.13.39**	**Nitric Oxide Synthase Inhibitor**	**8.86E-28**
**Phospholipase A2**	**3.1.1.4**	**Phospholipase A2 Inhibitor**	**9.82E-26**
Diaminopimelate epimerase	5.1.1.7	Nitric Oxide Synthase Inhibitor	2.43E-24
Membrane dipeptidase	3.4.13.19	Nitric Oxide Synthase Inhibitor	2.81E-23
*3-alpha(or 20-beta)-hydroxysteroid dehydrogenase*	*1.1.1.53*	*Aromatase Inhibitor*	*1.51E-22*
Sterol O-acyltransferase	2.3.1.26	Adenosine (A2) Agonist	4.95E-22
Hydroxymethylglutaryl-CoA reductase (NADPH)	1.1.1.34	Adenosine (A2) Agonist	4.95E-22
IMP dehydrogenase	1.1.1.205	Adenosine (A1) Agonist	8.98E-17
ATP-citrate (pro-S-)-lyase	4.1.3.8	Adenosine (A2) Agonist	1.83E-15
Glutamate–cysteine ligase	6.3.2.2	Nitric Oxide Synthase Inhibitor	2.71E-11
Dopamine-beta-monooxygenase	1.14.17.1	Adrenergic (beta1) Agonist	3.81E-11
*Lanosterol synthase*	*5.4.99.7*	*Squalene Synthase Inhibitor*	*1.38E-10*
Nucleoside-diphosphate kinase	2.7.4.6	P2T Purinoreceptor Antagonist	2.76E-10

a Exact matches (the enzyme is the canonical target of the best MDDR hit) are shown in **bold type**, pathway matches (the enzyme shares the same pathway as the canonical target of the best MDDR hit) are shown in *italic* type, and enzymes not in the same pathway as the canonical target are shown in regular type.

Other links recovered off-pathway hits, which often reflect known polypharmacology that is well-documented. For example, the glycinamide ribonucleotide formyltransferase (GART) inhibitor drug set hits both the GART reaction set (E = 1.55×10^−82^) and the off-pathway but pharmacologically related antifolate target dihydrofolate reductase (DHFR) (E = 1.02×10^−134^). Other off-pathway hits reflect biological connections, or physical connections, between targets. For example, the adenosine deaminase reaction set links to the A_1_ adenosine receptor agonist drug set (E = 7.69×10^−159^) (**Table 1**) capturing the known interaction between A_1_ adenosine receptors and adenosine deaminase on the cell surface of smooth muscle cells [33]. Considering only the stringent case of exact matches based on EC numbers, a Mann-Whitney rank-sum test (also referred to as the U-test) shows that the expectation values for links between reaction sets and drug sets of known drug target enzymes were significantly better than the expectation values for links to reaction sets of non-target enzymes, i.e., 62 known enzyme targets were recovered in a background of 4,920 non-target “other” enzymes at a statistical significance of P = 2.01×10^−6^.

In addition to recapitulating many known drug-target interactions, the links identified by these comparisons also suggest new hypotheses about drug-target interactions. One such new prediction involves the phospholipase A2 (PLA2) inhibitor drug class. The substrates and products of PLA2 recapitulate its known link to the PLA2 inhibitor drug set (E = 9.82×10^−26^), however, the sterol esterase reaction returns an even better score against the PLA2 inhibitor set (E = 3.18×10^−44^) (**Table 1**). Although this predicted pharmacological relationship has, to our knowledge, not been previously documented, the result is consistent with the known biological relationship between PLA2 and sterol esterase. Both enzymes are secreted by the pancreas and require phosphatidylcholine hydrolysis to facilitate intestinal cholesterol uptake [34]. Thus, this link suggests that therapeutic agents directed against PLA2 may also inhibit sterol esterase, perhaps even more strongly than their intended target.

### Human drug “effect-space” maps detail interactions between drug classes and enzyme target*s*

**Figure 3:**
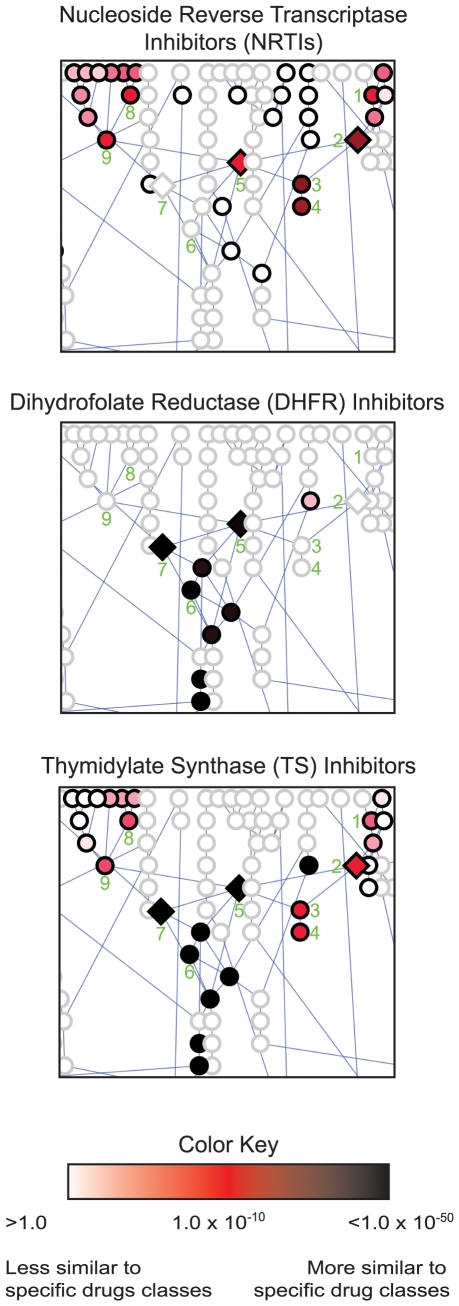
Effect-space map showing chemical similarity between specific drug classes and metabolites in human folate and pyrimidine biosynthesis. Each node represents one reaction set – the substrates and products of a single human metabolic reaction. Edges connect the reactions in the canonical pathway as annotated in HumanCyc [35]. As given in the color key, each reaction is colored according to the expectation value indicating the strength of similarity between that target reaction set and the respective MDDR drug set. Diamond shaped nodes indicate reactions catalyzed by enzymes annotated as known drug targets in the MDDR; circles indicate reactions catalyzed by enzymes not annotated as targets. Reaction key: 1. Deoxyuridine kinase 2. Thymidine kinase 3. Thymidylate kinase 4. Deoxythymidine diphosphate kinase 5. Thymidylate synthase (TS) 6. Methylene tetrahydrofolate reductase 7. Dihydrofolate reductase (DHFR) 8. Deoxyuridine diphosphate kinase 9. Deoxyuridine triphosphate diphosphatase.

To present links between small molecule metabolites and drugs in the context of their known (and potential) metabolic targets, metabolic “effect-space” maps for currently marketed drugs were generated for each of the 246 drug classes investigated in this work. These maps enable visualization of the chemical similarities between drugs and metabolites painted onto human metabolic pathways, illustrating potential interactions between an individual drug class and specific metabolic enzymes in humans. Examples include the nucleoside reverse transcriptase, dihydrofolate reductase, and thymidylate synthase inhibitors which target pyrimidine nucleotide metabolism and biosynthesis of the essential coenzyme folate (**Figure 3** **and** **Table 2**). Using the canonical human metabolic pathways from HumanCyc [35], a subset of the BioCyc [12] database collection, reactions in each metabolic network have been colored according to their similarity to known drug classes (**Figure 3**). While **Table 1** presents only the top link for each of 62 enzyme targets in MetaCyc against the 246 MDDR drug classes, the networks in **Figure 3** detail all significant hits for selected drug classes against the pyrimidine and folate pathways. Interactive versions of these maps, one for each of the 246 drug classes included in our analysis, are available online (see below).

**Table 2:** Links between selected drug classes and top ranked metabolic reactions.

**Rank**	**Thymidylate Synthetase (TS) Inhibitor**	**E-value**
1	Dihydrofolate reductase (DHFR)	1.96E-123
2	Methyltetrahydrofolate-corrinoid-iron-sulfur protein methyltransferase	3.58E-102
3	Methionyl-tRNA formyltransferase	1.97E-99
4	Methylenetetrahydrofolate reductase	2.67E-86
5	Thymidylate synthase (TS)	2.54E-75
6	Formate-tetrahydrofolate ligase	1.44E-74
7	Dihydrofolate synthetase	1.35E-70
8	Aminomethyltransferase	7.13E-63
9	5-methyltetrahydrofolate-homocysteine S-methyltransferase	2.80E-62
10	Phosphoribosylaminoimidazolecarboxamide (AICAR) formyltransferase	1.50E-60
11	Phosphoribosylglycinamide formyltransferase (GART)	1.50E-60
**Rank**	**Dihydrofolate Reductase (DHFR) Inhibitor**	**E-value**
1	Dihydrofolate reductase (DHFR)	1.46E-82
2	Methyltetrahydrofolate-corrinoid-iron-sulfur protein methyltransferase	2.84E-75
3	Methylenetetrahydrofolate reductase	6.01E-73
4	Methionyl-tRNA formyltransferase	7.00E-66
5	Aminomethyltransferase	6.90E-55
6	Formate-tetrahydrofolate ligase	6.15E-49
7	Thymidylate synthase (TS)	1.91E-48
8	5-methyltetrahydrofolate-homocysteine S-methyltransferase	2.60E-45
9	3-methyl-2-oxobutanoate hydroxymethyltransferase	2.68E-44
10	Glycine decarboxylase	2.68E-44
11	Glycine hydroxymethyltransferase (SHMT)	2.68E-44
12	Dihydrofolate synthetase	9.65E-42
13	Phosphoribosylaminoimidazolecarboxamide (AICAR) formyltransferase	2.21E-39
14	Phosphoribosylglycinamide formyltransferase (GART)	2.21E-39
**Rank**	**Nucleoside Reverse Transcriptase Inhibitor (NRTI)**	**E-value**
1	Thymidylate kinase	7.48E-28
2	Thymidine kinase	3.48E-26
3	Deoxythymidine diphosphate kinase	1.54E-24
4	Ribonucleoside-triphosphate reductase	2.88E-14
5	Deoxyuridine triphosphate pyrophosphatase	5.60E-12
6	Deoxyuridine kinase	1.14E-11
7	Deoxyuridine diphosphate kinase	1.45E-11
8	Thymidylate synthase (TS)	5.68E-11

It has previously been shown that chemical similarity between known drugs often suggests novel drug-target interactions [5]–[7],[14]. Consistent with these observations, effect-space maps such as those shown in **Figure 3** can also be used to exploit chemical similarities between drugs and metabolites to indicate potential routes of drug metabolism and toxicity [3],[11],[36],[37]. For example, the nucleotide reverse transcriptase inhibitors (NRTIs) used in HIV therapy are administered as pro-drugs. The effect-space map reflects this route of NRTI metabolism leading to viral inhibition. The top three hits yielded by the NRTI drug set queried against human metabolism – thymidine kinase (E = 3.48×10^−26^), thymidylate kinase (E = 7.48×10^−28^), and deoxythymidine diphosphate kinase (E = 1.54×10^−24^) (**Figure 3** reaction numbers 2, 3, and 4; additional results in **Table 2**) – successively phosphorylate the NRTI pro-drugs into the pharmacologically active NRTI triphosphates [38],[39]. The viral reverse transcriptase enzyme then incorporates the fully phosphorylated NRTIs into the growing DNA strand, thereby terminating transcription of the viral DNA. In this example, considerable toxicity mitigates the therapeutic value of inhibiting viral DNA transcription since the phosphorylated NRTIs directly inhibit human nucleotide kinases and mitochondrial DNA pol-γ. They also may be incorporated by pol-γ into the growing human mitochondrial DNA strand, and once incorporated are inefficiently excised by DNA pol-γ exonuclease [40]. Thus, the effect-space map illustrates both the route of metabolism and a mechanism of toxicity for NRTIs in humans.

**Figure 4:**
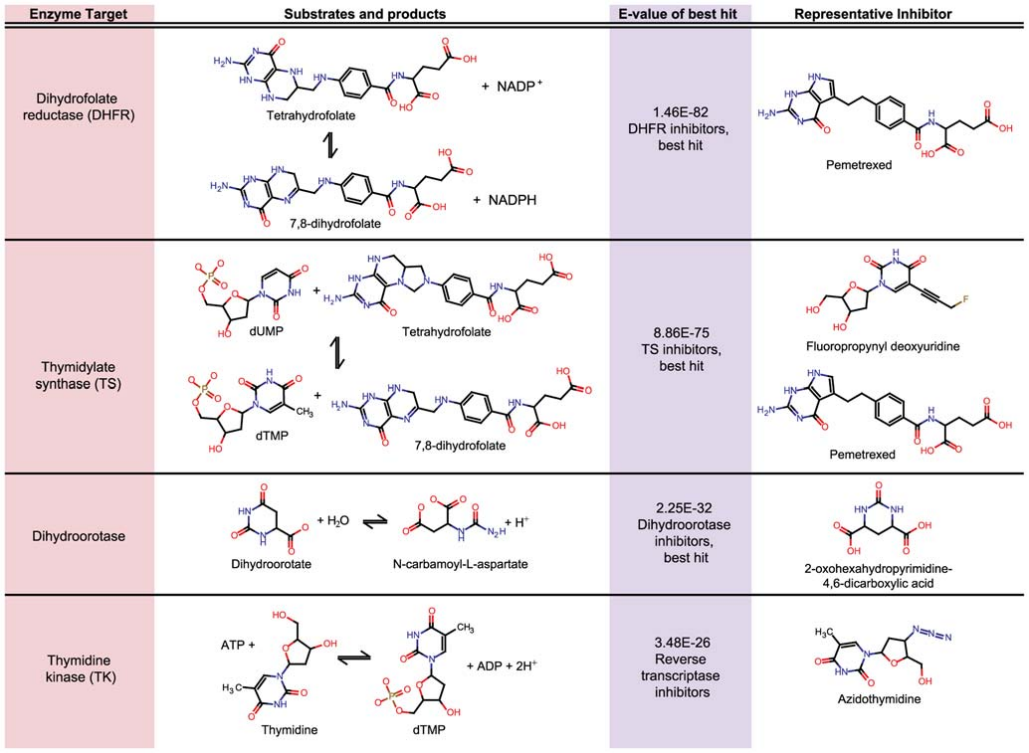
Selected links between MDDR drug classes and human folate and pyrimidine metabolism.

Drug effect-space maps also offer a broad glimpse of potential human metabolic interactions predicting new “polypharmacology”. From the ligand perspective, “drug polypharmacology” refers to a single drug or drug class that hits multiple targets. For example, dihydrofolate reductase (DHFR, reaction number 7 in **Figure 3**) uses NADPH to reduce 7,8-dihydrofolate to tetrahydrofolate. Antifolate drugs inhibit DHFR, and, as expected, the DHFR drug set recovers the DHFR reaction substrates and products as the top similarity hit in human metabolism (E = 1.46×10^−82^) (**Figure 3**, **Table 2**, **Figure 4**). However, at least 20 other reactions also use folate coenzymes in human metabolism [41]–[43]. Accordingly, SEA finds additional links between the DHFR drug set and established antifolate targets outside the pyrimidine and folate biosynthesis pathways such as serine hydroxymethyltransferase (SHMT, E = 2.68×10^−44^), phosphoribosyl-aminoimidazole-carboxamide formyltransferase (AICAR transformylase, E = 2.21×10^−39^), and phosphoribosyl-glycinamide formyltransferase (GART, E = 2.21×10^−39^) (**Table 2**). The effect-space maps in **Figure 3** illustrate the results from **Table 2** and **Figure 4** in a single view, illustrating drug polypharmacology with respect to critical metabolic pathways.

Alternatively, from the target perspective, “target polypharmacology” may refer to a single target being modulated by multiple classes of drugs. For instance, thymidylate synthase (TS) is another classic antifolate target that uses a folate coenzyme to methylate deoxyuridine phosphate, generating deoxythymidine phosphate [44]–[47]. As expected, the TS reaction links to known antifolate drug classes such as GART inhibitors (E = 4.76×10^−73^) and DHFR inhibitors (E = 1.91×10^−48^) (**Table 3** **and** **Figure 4**). However, TS is also effectively inhibited by uracil analogs such as fluoropropynyl deoxyuridine, which is not a folate, but rather a pyrimidine analog. Accordingly, the TS reaction also links to reverse transcriptase inhibitors, which include fluoropropynyl deoxyuridine and additional pyrimidine analogs such as azidothymidine (AZT) (E = 5.68×10^−11^) (**Figure 4**). The target polypharmacology of the thymidylate synthase enzyme is mirrored by the drug polypharmacology of the thymidylate synthase inhibitors. The TS inhibitors link not only to the reactions of deoxyribonucleotide biosynthesis including thymidylate synthase (E = 2.54×10^−75^), but also the GART (E = 1.50×10^−60^) and DHFR (E = 1.96×10^−123^) reactions (**Figure 3** **and** **Table 2**). Thus, SEA recapitulates the known polypharmacology of TS. Effect-space maps illustrate and clarify these pharmacological relationships.

**Table 3:** Links between selected metabolic reactions and top ranked drug classes.

**Rank**	**Thymidylate Synthetase (TS) Reaction**	**E-value**
1	Thymidylate synthase inhibitor (TS)	2.54E-75
2	Glycinamide ribonucleotide formyltransferase inhibitor (GART)	4.76E-73
3	Thymidine kinase inhibitor (TK)	1.18E-62
4	Dihydrofolate reductase inhibitor (DHFR)	1.91E-48
5	Folylpolyglutamate synthetase inhibitor	2.27E-31
6	Nucleoside reverse transcriptase inhibitor (NRTI)	5.68E-11
**Rank**	**Dihydrofolate Reductase (DHFR) Reaction**	**E-value**
1	Glycinamide Ribonucleotide Formyltransferase Inhibitor	1.02E-134
2	Thymidylate Synthetase Inhibitor	1.96E-123
3	Dihydrofolate Reductase Inhibitor	1.46E-82
4	Folylpolyglutamate Synthetase Inhibitor	3.15E-62

### Species-specific effect-space maps for pathogens and model organisms

**Figure 5:**
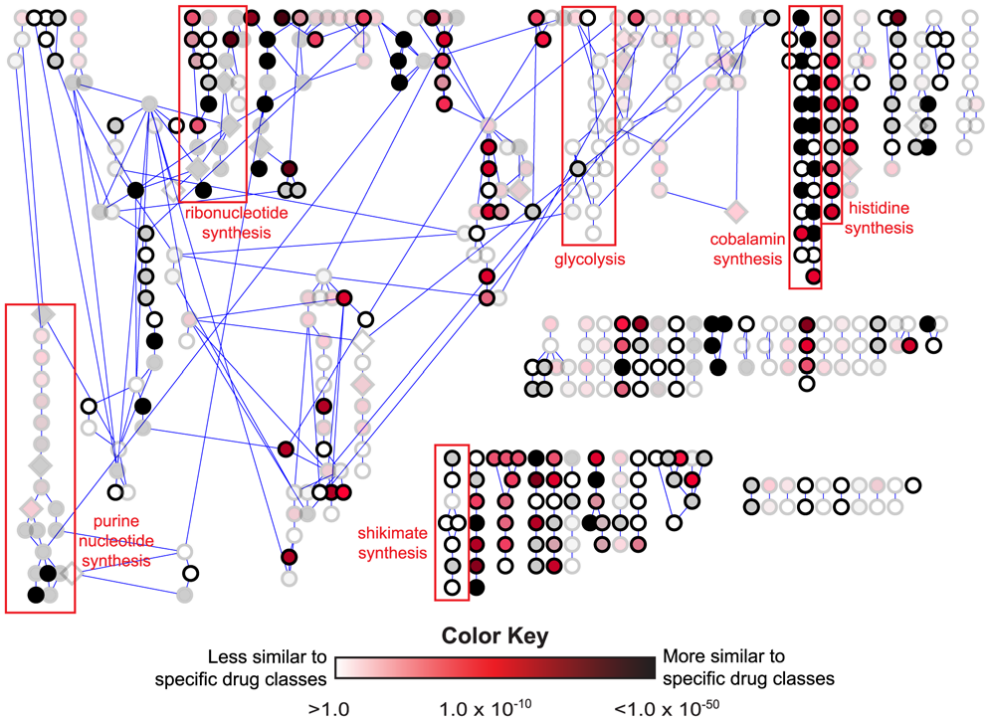
Effect-space map showing chemical similarity between drugs and metabolites in MRSA. Canonical pathway representation of methicillin-resistant *Staphylococcus aureus* (MRSA) [12] small molecule metabolism colored by expectation value of the best hit against MDDR. Reactions that are also present in humans have been faded. Layout based upon the Cytoscape 2.5 y-files hierarchical layout. Edge lengths are not significant. For ease of viewing, reactions are not labeled but can be identified in the interactive versions of the maps available at the online resource.

The great diversity of metabolic strategies, pathways, and enzymes present in humans, model organisms, and pathogenic species presents both opportunities and significant barriers to drug discovery. To address these issues, species-specific effect-space maps were created for each of 385 organisms from the BioCyc Database Collection. Target reactions existing in common and differentially between each of these species and humans are shown in these metabolic maps. As with the human effect-space maps, this set of maps is available in interactive form online. To show how these maps may be used to provide a context for drug discovery, MRSA is used as an example (**Figure 5**). The global view of drugs and metabolism provided by this species-specific map illustrates some of the daunting challenges to the selection of tractable metabolic drug targets in this organism.

As described for **Figure 3**, each node in the MRSA network in **Figure 5** represents one reaction set, the substrates and products of a single metabolic reaction. Edges connect the reactions according to canonical BioCyc MRSA pathways. Each reaction in the network has been colored according the expectation value of the best link between the reaction set and any of the 246 MDDR drug sets. Lighter colored nodes have higher expectation values indicating less drug-like reaction sets, while darker colored nodes indicate more drug-like reaction sets. To provide therapeutic context, reactions that are also present in human metabolism have been faded, indicating that drug sets targeting these enzymes in MRSA may have the undesirable potential to inhibit the human enzymes as well. As with the other organisms represented in our online maps, most reactions in the MRSA subset have little chemical similarity to any MDDR drug set. Although 74% of the 469 MRSA metabolic reactions have measurable similarity to at least one MDDR drug set, only 36% of these links had expectation values of E = 1.0×10^−10^ or better. Several complete pathways of diverse chemical classes, including shikimic acid, phospholipid, peptidoglycan, teichoic acid, and molybdenum cofactor biosynthesis, lack links to any drug set at all. Only 18 of the 469 MRSA metabolic reactions are already known to be drug targets in MDDR. Fourteen of these are represented in **Figure 5** (as diamonds), but all 18 of these also appear in humans. Enzymes that catalyze these reactions in humans would likely be vulnerable to inhibitors developed against these MRSA targets, putting those drugs at risk for toxicity.

**Figure 6:**
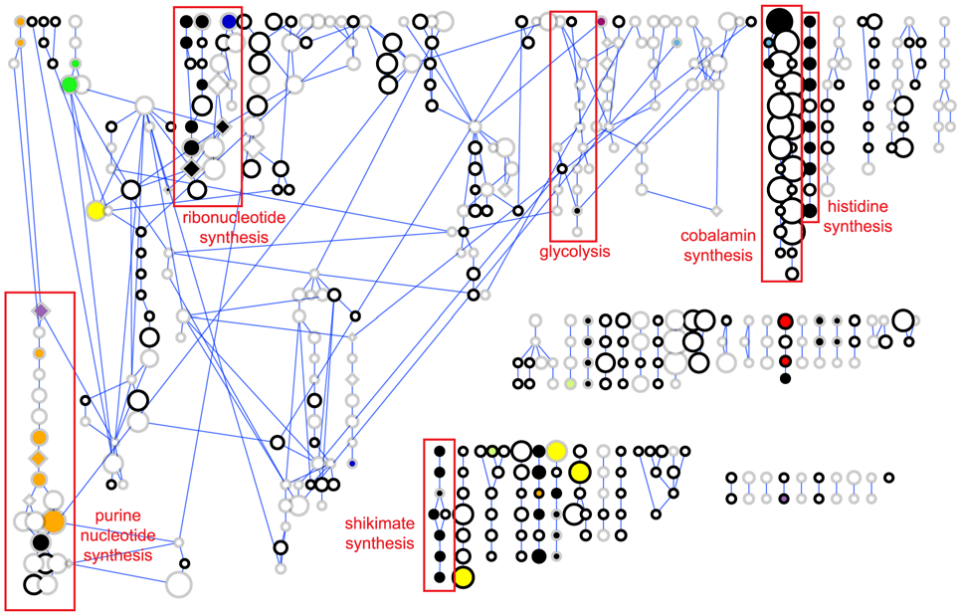
Essential and synthetic lethal map of MRSA metabolism. Canonical pathway representation of methicillin-resistant *Staphylococcus aureus* (MRSA) small molecule metabolism colored by essentiality and synthetic lethality of reactions. Key: black = essential reaction; other colors = synthetic lethal reaction pairs; node size = similarity to top MDDR hit (bigger is more drug-like); diamond shape = MDDR drug target; faded border = human reaction.

**Figure 6** illustrates how additional information can be used to further filter potential metabolic targets by painting additional biological or genomic data onto a species-specific map. Since successful modulation of a target may not alone be sufficient to kill a pathogen due to the presence of redundant pathways for the formation of critical metabolites, integration of such additional information into a metabolic map may provide added value in addressing the multi-dimensional challenges of drug discovery. Flux balance analysis of metabolic networks was used by several of the authors of this work to identify essential enzymes and metabolites required for the formation of all necessary biomass components for 13 strains of *Staphylococcus aureus* including the methicillin-resistant N315 strain (MRSA) [48]. Using these results, 39 essential reactions and 19 synthetic lethal reaction pairs could be mapped to our dataset (**Figure 6**), highlighting those reactions for which inhibition is most likely to result in the death of the organism. Several of these reactions have been successfully targeted by currently marketed drugs, such as the previously discussed antifolate targets DHFR (E = 1.02×10^−134^), thymidylate synthase (E = 2.54×10^−75^), and dihydrofolate synthase (E = 1.35×10^−70^). This retrospective result illustrates the potential of such additional information in enriching for targets and drug chemistry that have been proven accessible. Other targets and pathways have not yet yielded successful drugs but are under investigation in MRSA or other pathogens, such as the shikimate pathway [49] in aromatic amino acid biosynthesis and the histidine biosynthesis pathway [50].

The combination of the essentiality data with the drug space mapping emphasizes the challenges to drug discovery against MRSA. Thus, while species-specific antifolates do exist, many antifolates such as methotrexate used in cancer therapy cause severe toxicity [43]. To avoid such toxicity, 14 of the 39 essential MRSA reactions that are also present in humans can be excluded from further consideration as drug targets in MRSA.

A compilation of all of the metabolic network maps generated in this study is available at http://sea.docking.org/metabolism. These include interactive versions of the human effect-space maps shown in **Figure 3**, one for each of the 246 MDDR drug classes analyzed in this work, and 385 species-specific maps such as that shown in **Figure 5**. The species-specific maps were generated from the BioCyc database public collection, a compendium of 385 model organisms and pathogens whose genomes have been sequenced and their metabolomes deciphered. Of these, 65 have been designated as Priority Pathogens by the National Institute of Allergy and Infectious Diseases (NIAID) and include *Bacillus anthracis*, *Brucella melitensis*, *Cryptosporidium parvum*, *Salmonella*, SARS, *Toxoplasma gondii*, *Vibrio cholerae*, and *Yersinia pestis* [51]. Browse and similarity search tools are also provided, allowing exploration of the metabolic reaction sets and current drug classes used in this work, as well as comparison to user-defined custom ligand sets. These interactive tools enable facile exploration between the vast biological data on potential metabolic drug targets in these organisms and the drug chemistry currently available to prosecute those targets.

## Discussion

A key product of this study is the construction of drug-metabolite correspondence maps that provide both a global view and a more contextual picture of predicted drug action in human metabolism than has been previously available. Several aspects of these maps deserve particular emphasis. First, despite the differences in physiochemical properties of most drugs and small molecule metabolites, numerous links arise between drugs and metabolism. Viewed in the context of metabolic networks, the pharmacological relationships predicted by these links can be readily interpreted in a way that is biologically sensible. Moreover, as shown by both the drug effect space maps and species-specific maps, our retrospective analyses confirm that biologically and pharmacologically significant connections can be recovered, capturing known polypharmacology and revealing the relevant chemotypes previously explored in drug development. The metabolome-wide exploratory tools provided with these map sets also enable a new way to interrogate the links between drugs and metabolism that will likely be useful for prediction of new targets and to indicate routes of drug metabolism and toxicity. Further, by integrating biological information such as essentiality and synthetic lethal analyses with the metabolic context, our approach allows users to focus evaluation of potential targets around specific types of data simply by painting the results on to metabolic maps.

With respect to the coverage of drug links across small molecule metabolism that this study provides, we note that the SEA method relies solely upon the chemical similarity of ligands to establish links between drug sets and reaction sets. Based on these links, and the biologically sensible connections shown in the results, we infer that a particular drug class may act on a certain target. However, drugs may also act against an enzyme active site without resembling the endogenous substrate, or by allosteric regulation at an entirely different site. The SEA method, as applied here to the substrates and products of metabolic reactions, does not capture these additional drug-target links. Other viable strategies are available for targeting metabolic enzyme active sites that use principles unrelated to the ligand-drug similarities that are the focus of our approach [52]–[55]. For instance, Tondi et al. designed novel inhibitors of thymidylate synthase that complemented the three dimensional structure of the active site. Five high-scoring compounds selected for testing were dissimilar to the substrate but bound competitively with it [55]. While many classical kinase inhibitors interact directly with the ATP binding site, imatinib (tradename Gleevec) represents a new generation of allosteric protein kinase inhibitors that alter the kinase conformation to prevent ATP binding. Other allosteric kinase inhibitors prevent the protein substrate from loading [52].

While a quantitative determination of the proportion of drug-target links that cannot be accessed by our approach is beyond the scope of this study, we can provide a rough estimate for the frequency of such cases based on the results reported in **Table 1**. Of the 62 known enzyme targets in MetaCyc, 42 (68%) the substrate/product metabolite sets show significant chemical similarity to at least one MDDR drug set, establishing a reasonable first pass estimate for the percentage of current enzyme targets accessible to this approach. Furthermore, 40% (2,044 of 5,056) of all MetaCyc reaction sets linked at E = 1.0×10^−10^ or better to MDDR, with each reaction linking to an average of just 2.8 MDDR drug sets. These results indicate broad and specific coverage of metabolism, and suggest that numerous additional enzyme targets may be accessible by the method presented here.

### Conclusion

Using the SEA method, we have shown that comparison between ligand sets representing MDDR drug classes and ligand sets representing the substrates and products of metabolic reactions yields statistically significant links between known drugs and enzyme targets. Because the method is based on chemical similarity and requires only information from these molecule sets rather than the sequence, structure or physiochemistry of the targets, this ligand-based approach is independent from, and complementary to, protein structure and sequence based methods. Our results also suggest the potential of this method for predicting previously unknown interactions between drug classes and metabolic targets, recovering routes of metabolism and toxicity in humans, and identifying potential drug targets (as well as challenges for target discovery) in emerging pathogens. Thus, by mapping the chemical diversity of drugs to small molecule metabolism using ligand topology, this work establishes a computational framework for ligand-based prediction of drug class action, metabolism, and toxicity.

## Methods

### 

#### Compound sets

All compounds, both drugs and metabolites, are represented using Daylight SMILES strings [29]. Sets comprised of isomers with unique compound names were retained, even though stereochemistry was later removed as part of the molecule fingerprinting process.

#### Ligand sets

Reaction sets were extracted from the 8.15.2007 release of MetaCyc based upon the substrates and products annotated to each reaction. Two filters were applied. First, the ten most common metabolites based on the number of occurrences in the MetaCyc metabolic network were removed: water, ATP, ADP, NAD, pyrophosphate, NADH, carbon dioxide, AMP, glutamate, and pyruvate. Second, each reaction set was required to include at least two unique compounds, as indicated by a MetaCyc or a MDDR unique compound id.

#### Drug sets

Drug sets were extracted from the MDDR, a compilation of about 169,000 drug-like ligands in 688 activity classes, each targeting a specific enzyme (designated by the Enzyme Commission (E.C.) number). The subset of this database for which mappings between enzymes and the MDDR drug classes were available was used. These mappings were based on a previous study that maps E.C. numbers, GPCRs, ion channels and nuclear receptors to MDDR activity classes [32]. Only sets containing five or more ligands were used. Salts and fragments were removed, ligand protonation was normalized and duplicate molecules were removed. Of the 688 targets in the MDDR, 97 were excluded as having too few ligands (<5), and another 345 targets were excluded because their definitions did not describe a molecular target, e.g., drugs associated only with an annotation such as “Anticancer” were not used. The remaining 246 enzyme targets were together associated with a total of 65,241 unique ligands, with a median and mean of 124 and 289 drug ligands per target. For further details, see Keiser et al. [6].

#### Set comparisons

All pairs of ligands between any two sets were compared using a pair-wise similarity metric, which consists of a descriptor and a similarity criterion. For the similarity descriptor, standard two-dimensional topological fingerprints were computed using the Scitegic ECFP4 fingerprint [56]. The similarity criterion was the widely used Tanimoto coefficient (Tc) [57]. For set comparisons, all pair-wise Tcs between elements across sets were calculated, and those scoring above a threshold were summed, giving a raw score relating the two sets. The Tanimoto coefficient threshold of 0.32 was determined according to a previously published method based upon fit to an extreme value distribution [6]. A model for random similarity similar to that used by BLAST [58] was used to generate expectation values (E) which are used to describe the strengths of relationships discovered using this protocol [6]. All scores reported here are based upon the background distribution and cutoff scores generated using the drug sets extracted from the MDDR collection. For further details, see Keiser et al. [6]. Network visualization was performed in Cytoscape 2.6.2 [59] using the γ-files hierarchical layout algorithm.

#### MRSA essentiality and synthetic lethal analysis

Essentiality and synthetic lethal data generated as described earlier [48]. Briefly, the metabolic network was reconstructed from the genome to include all reactions that have an active flux The essentiality of a given enzyme was then assessed by the effect of the removal of that enzyme on biomass production. Similarly, synthetic lethal pairs can be identified by systematic pairwise deletion of enzymes and recalculation of biomass production in an ideally rich medium.

## Supporting Information

Dataset S1 MetaCyc reaction sets
(0.47 MB TXT)

Dataset S2 SMILES describing the molecular strucutre of MetaCyc reaction substrates and products
(0.25 MB TXT)

Dataset S3 MDDR drug sets
(0.51 MB TXT)

Dataset S4 SMILES describing the molecular structure of MDDR ligands.
(4.45 MB TXT)

Dataset S5 E-values for links between MDDR drug sets and MetaCyc reaction sets
(3.12 MB CSV)
